# Spouses’ faces are similar but do not become more similar with time

**DOI:** 10.1038/s41598-020-73971-8

**Published:** 2020-10-12

**Authors:** Pin Pin Tea-makorn, Michal Kosinski

**Affiliations:** 1 Department of Electrical Engineering, Stanford University, 350 Jane Stanford Way, Stanford, CA 94305 USA; 2 Graduate School of Business, Stanford University, 655 Knight Way, Stanford, CA 94305 USA

**Keywords:** Computational biology and bioinformatics, Data mining, Machine learning, Psychology, Human behaviour

## Abstract

The widely disseminated convergence in physical appearance hypothesis posits that long-term partners’ facial appearance converges with time due to their shared environment, emotional mimicry, and synchronized activities. Although plausible, this hypothesis is incompatible with empirical findings pertaining to a wide range of other traits—such as personality, intelligence, attitudes, values, and well-being—in which partners show initial similarity but do not converge over time. We solve this conundrum by reexamining this hypothesis using the facial images of 517 couples taken at the beginning of their marriages and 20 to 69 years later. Using two independent methods of estimating their facial similarity (human judgment and a facial recognition algorithm), we show that while spouses’ faces tend to be similar at the beginning of marriage, they do not converge over time, bringing facial appearance in line with other personal characteristics.

## Introduction

What predisposes two people to form and maintain a long-term romantic relationship is a fundamental question with critical consequences for the individuals involved, their families, and entire societies. While we do not yet have a satisfactory answer, one thing is clear: Romantic partners tend to be similar in a wide range of characteristics, ranging from physical and physiological to demographics and psychological^1^. Long-term romantic partners have been shown to be similar in terms of height, weight, health, diet, age, physical attractiveness, education, ability, intelligence, psychological well-being, personality, attitudes, values, religion, social class, ethnicity, lifestyle, and many other traits^2–8^.

What drives romantic partners’ similarity? Two sets of mechanisms have been proposed to explain it. First, partners may be *similar from the outset of their relationship* due to homophily (i.e., preference for similar others)^9,10^, the mechanics of the dating market (e.g., having to settle for a partner with a similar level of attractiveness)^11^, or social homogamy (i.e., being surrounded—socially and geographically—by similar others)^8^. Second, partners may *become more similar* with time due to repeated interactions, synchronized routines, shared environment^12,13^, and/or attrition (i.e., less similar couples breaking up, thus boosting the average similarity of the surviving ones)^14,15^. Although both sets of mechanisms seem plausible, empirical research consistently suggests that couples are similar to begin with but do *not* become any more similar with time. Long-term couples, for example, exhibit similarity patterns parallel to new couples^16^, and are no more similar in terms of attitudes, values, intelligence, personality, psychological well-being, and interests^3,4,17–19^. Also, partners’ personality and interests are similar even before they met (online) for the first time^20^. These and other analogous findings led most scholars to conclude that shared life experiences and circumstances play a significant role in maintaining, rather than increasing, couples’ initial similarity^2,21^.

There is, however, one trait that does not seem to follow this general pattern: facial appearance. In their seminal paper, Zajonc, Adelmann, Murphy, and Niendenthal^12^ showed that spouses’ faces were not similar at the outset of marriage but became more similar with time. Moreover, they found that the degree of convergence positively correlated with couples’ ratings of marriage quality. Their *convergence in physical appearance hypothesis* posits that as long-term partners tend to occupy the same environments, engage in the same activities, eat the same food, and mimic each other’s emotional expressions—and as these factors can also influence facial features—spouses’ facial appearances should converge with time. For example, if the partners smile a lot—and make each other smile—they should co-develop similar wrinkle patterns (smile lines)^22^.

Importantly, Zajonc et al.’s reasoning^12^—that appearance converges as a function of shared actions and environment, and emotional mimicry—should apply to other personal characteristics as well. How does one reconcile the convergence in facial appearance with the lack thereof in the context of virtually all other traits, such as interests, personality, intelligence, attitudes, values, and well-being? A closer look at the literature reveals that while the convergence in physical appearance hypothesis is one of the tenets of current psychological science and has been widely disseminated through textbooks^23^, books^24,25^, and landmark papers^26,27^, it has virtually no empirical support. Zajonc et al.’s study^12^, while elegantly designed, was based on an extremely small sample of 12 married heterosexual couples. Furthermore, its findings have never been replicated. Two other studies occasionally cited in support of facial convergence (Hinsz^28^ and Griffith and Kunz^29^) neither tested this hypothesis nor provided any support for it. Both studies presented evidence for *facial homogamy*, i.e., spouses’ tendency to have similar faces, but provided no support for the increase in facial resemblance over time. Hinsz^28^ found that romantic partners’ faces were more similar than those of random pairs of men and women, yet couples married for 25 years were no more similar than recently engaged ones. Griffith and Kunz^29^ showed that student raters could match spouses’ faces at a level above chance, yet found “no significant trend in growing to look alike as persons live together as husband and wife” (p. 453).

In this work, we aim to validate the physical convergence hypothesis in a large sample (n = 517) of white married heterosexual couples (we were unable to find a large enough sample of homosexual and non-white couples to allow for a meaningful analysis). Two approaches to measuring facial similarity were used: human judges and a modern facial recognition algorithm. Both approaches showed that while spouses’ faces were similar at the outset of their marriage, they did not converge over time.

## Methods

The study has been reviewed and approved by Stanford University’s IRB. All methods were carried out in accordance with relevant guidelines and regulations. The preregistration documents can be found at https://aspredicted.org/2fh78.pdf. The Supplementary Information contains the list of and rationale for the post-registration changes to the study design. The materials, data, and code used to compute the results are available at https://osf.io/ekwm7.

### Facial images

The facial images of 517 couples were collected from public online sources: 392 newspaper wedding anniversary announcements downloaded from https://www.newspaperarchive.com, 102 Google Search results, and 23 public profiles from Ancestry.com (a genealogy website). Two facial images of each spouse were collected: one taken within 2 years of the wedding, and one taken 20 to 69 years later (the marriage dates and dates on which the photos were taken were extracted from their captions; the average marriage length was 49 years).

**Figure 1:**
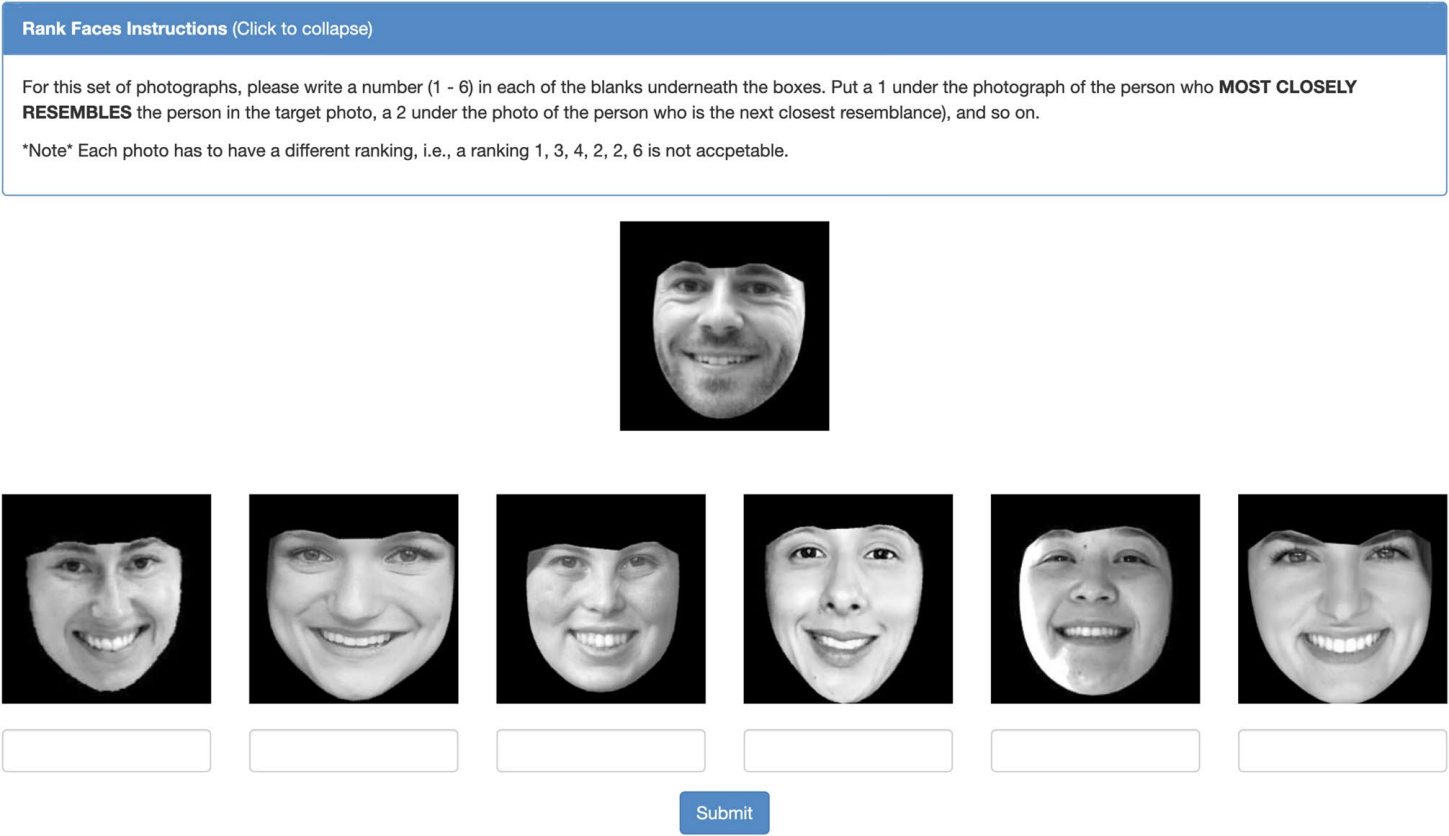
An example stimulus set (to protect participants’ privacy, we used photos of our colleagues. Their informed consent for publication was obtained).

Images were processed using Face++ (https://www.faceplusplus.com)—a widely used facial recognition software—to detect facial outlines and head orientation, and to approximate individuals’ age (see Supplementary Fig. S1 for age distribution). We only included images containing faces larger than 120 × 120 pixels and with an absolute value of yaw and pitch below  55° and 24°, respectively. The images were converted into grayscale and cropped around the face to remove the background and non-facial details. Their brightness was corrected using the “auto-adjust colors” function in IrfanView 4.5. The faces were rotated to the vertical position and resized to 224 × 224 pixels (see Fig. 1).

### Stimulus sets

Faces were arranged into 2068 unique stimulus sets (517 couples × two spouses × two time points: at the beginning of the marriage and 20 to 69 years later) following the procedure from Zajonc et al.^12^ Each face (*target*) was matched with faces of six other people of the opposite sex (*alternatives*): the target’s spouse and five random others from our dataset. To control for the effect of age and eyewear, the alternatives had the same eyewear status (glasses or no glasses) and similar approximated age (+ /− 5 years) as the spouse. An example stimulus set is presented in Fig. 1.

### Human judges and rankings

Judges (n = 153; from the U.S.), recruited on Amazon Mechanical Turk (AMT; an online crowdsourcing marketplace), were instructed to rank alternative faces from the most (1) to the least (6) *closely resembling* the target face (see Fig. 1). Ten rankings were obtained for each stimulus set. The spouses’ perceived similarity at a given point in time (at the beginning of the marriage and later) was computed by averaging their ranks across two stimulus sets pertaining to them (husband as a target and wife as a target). The resulting scale ranged from 1 (all judges perceived them as most similar) to 6 (all judges perceived them as least similar). If there was no link between being married and similarity, the spouses’ average rank should equal 3.5. The use of a relative (i.e., ranking) rather than absolute (e.g., Likert scale) measure of facial similarity enabled controlling for the possibility that people’s faces may generally become more (or less) similar with time as they age.

Additionally, following Zajonc et al.’s^12^ original design, a separate sample of 117 judges recruited on AMT were asked to rank alternatives in terms of their *likelihood to be married to the target* (the same stimulus as presented in Fig. 1 was used, with “closely resembles” replaced with “likely to be married to”).

### Facial recognition algorithm and rankings

An alternative set of results was produced using VGGFace2^30^, a widely used facial recognition deep neural network that was shown to outperform humans in judging facial similarity^31^. Facial recognition algorithms convert faces into numerical vectors (*face descriptors*) capturing facial features and compare those vectors across images: The more similar the vectors, the more likely they are to represent the same face. As facial recognition algorithms are aimed at recognizing people across images taken at different times, with different devices, from different angles, and in different circumstances, they tend to capture features that remain stable across age and context, such as facial morphology and complexion. They are as unaffected as possible by transient features such as aging, facial expression, head orientation, hairstyle, and image properties such as background and lighting^32^. Consequently, they are well suited to the task of quantifying the similarity between faces, while controlling—as much as possible—for transient features.

Following the standard procedure used in facial recognition, cropped facial images were converted into 2048-value-long face descriptors using VGGFace2 in SE-ResNet-50 architecture and L2-normalized. Next, for each stimulus set, the cosine similarity between face vectors of the target and each alternative face was computed. The alternative faces were ranked from the most (1) to the least (6) *closely resembling* the target face (i.e., the same ranking scale as for human judges).

### Statistical analyses

The average similarity ranks of spouses’ faces were compared with the chance value (3.5) using one sample two-tailed t-test to detect homogamy. Paired two-tailed t-tests were used to compare the similarity of spouses’ faces at the beginning of marriage and later to detect the convergence in facial appearance. The average Kendall rank correlation between two randomly selected rankings for each stimulus set was used to measure inter-rater reliability.

## Results

**Figure 2:**
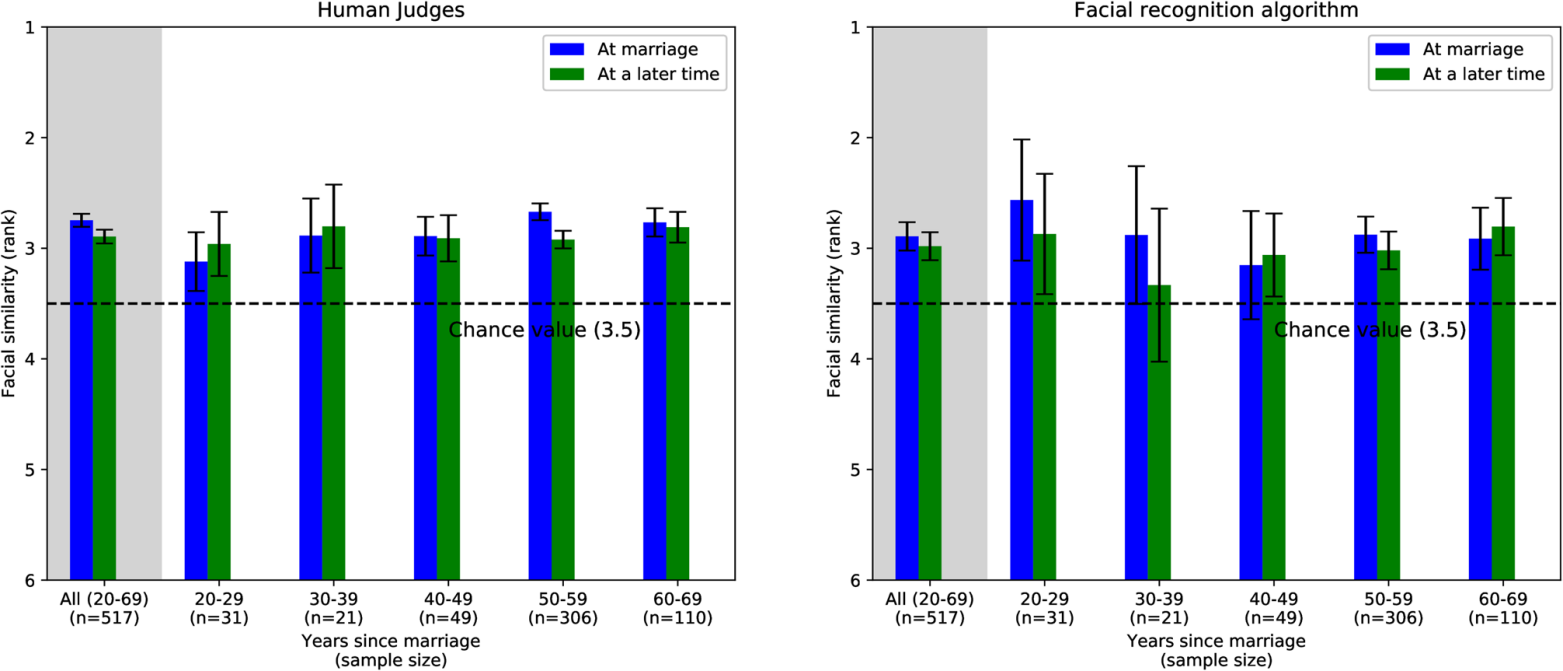
The average facial similarity of the spouses at marriage and 20 to 69 years later. Error bars represent 95% confidence intervals (also see Supplementary Table S1 online).

Figure 2 shows the similarity ranks produced by human judges (left panel) and VGGFace2 (right panel) at the time of marriage (blue bars) and 20 to 69 years later (green bars). The combined results for all age groups are shown on the gray background. Consistent with the previous studies^28,29,33–35^, we found evidence of homogamy, or spouses’ tendency to have similar faces. At the time of marriage, their average rank was significantly lower than 3.5 (i.e., the rank expected if the alternatives were ranked randomly): 2.75 (95% CI = [2.69, 2.81], one sample t-test t = − 25.08, two-tailed p < 0.001, n = 517) for human judges; and 2.89 (95% CI = [2.76, 3.02], one sample t-test t = − 9.32, two-tailed p < 0.001, n = 517) for VGGFace2.

However, we did not find evidence for the convergence in physical appearance hypothesis: Spouses’ faces did *not* become more similar with time. In fact, according to human judges, spouses’ faces became slightly less similar with time (paired t-test; t = − 3.70, two-tailed p < 0.001, n = 517), though the difference in the rankings was relatively small (Δ = 0.15, 95% CI = [0.07, 0.22]) and was not replicated in the VGGFace2 analysis. The same results were obtained when analyzing data separately for couples married for different lengths of time (Fig. 2): Spouses’ faces tended to be similar but did not become more similar with time, regardless of the time span between the first and the second set of pictures.

Importantly, judgments’ reliability did not vary with subjects’ age or time when the picture was taken: There was no significant difference between the inter-rater reliability for pictures taken at the time of marriage and later (Kendall τ_marriage_ = 0.165; 95% CI = [0.161, 0.168] and τ_later_ = 0.161; 95% CI = [0.157, 0.165]; τ_marriage_ − τ_later_ = 0.004, 95% CI = [− 0.001, 0.009], two-tailed p = 0.95). This indicates that the judges were as consistent when ranking the similarity of faces of young people (taken several decades ago) as the faces of older people (taken more recently).

As in the context of facial similarity (and contrary to Zajonc et al.’s^12^ findings), there were also no significant differences in judges’ ratings of spouses’ likelihood to be married between facial images taken at the time of marriage and later (paired t-test; t = − 1.51, two-tailed p = 0.13, n = 517; see Supplementary Table S2 for details).

## Discussion

We do not find support for the widely disseminated convergence in physical appearance hypothesis: Spouses’ faces are similar but do *not* converge with time. This brings facial appearance in line with other traits—such as interests, personality, intelligence, attitudes, values, and well-being—which show initial similarity but do not converge over time^2^.

This study has several limitations. First, we used publicly available images and thus could not control for variance in image properties and self-presentation (such as grooming, facial expression, or biases in selecting images to be publicly shared online). Yet, according to the convergence in physical appearance hypothesis, these factors should amplify the convergence rather than obscure it. Spouses’ tendency to occupy the same environments, engage in the same activities, eat the same food, and—in particular—mimic each other’s emotional expressions should result in convergence in their self-presentation behaviors, and thus more (and not less) similar public facial images. Second, we did not record or control for judges’ age and ethnicity and thus the extent to which their judgments might have been affected by the own-age^36^ and own-ethnicity^37^ biases (people’s lower sensitivity when judging the similarity of faces of other ages and ethnic groups). Yet, while the own-ethnicity bias could add noise to our measurements, it is unlikely to moderate the change in similarity over time, as participants’ ethnicity was constant. Also, while the U.S. AMT workers tend to be young^38^, they were as good at ranking the similarity of faces of young people (taken several decades ago) as the faces of older people (taken more recently). Furthermore, those and other risks to the judges’ accuracy were counterbalanced by the use of two independent measures of facial similarity (human judges and VGGFace2) and the relatively large sample size, enabling the detection of a change in human rankings as small as Δ = 0.17 (with 80% power, α = 0.001), an equivalent of one in six judges increasing a spouse’s rank by just one position. Finally, the validity of our approach and dataset are supported by the successful replication of the well-established effect of people’s tendency to marry similar others (i.e., homogamy).

While the *rejection* of the convergence in physical appearance hypothesis is surely not as exciting or as cite-worthy as its counterfactual, it solves one of the major conundrums of psychological science and brings us closer to understanding factors predisposing people to form and maintain long-term romantic relationships.

## Supplementary information

Supplementary Information.

Supplementary Figure S1.

## References

[1] Buss DM (1985). Human mate selection: Opposites are sometimes said to attract, but in fact we are likely to marry someone who is similar to us in almost every variable. Am. Sci..

[2] Luo S (2017). Assortative mating and couple similarity: Patterns, mechanisms, and consequences. Soc. Pers. Psychol. Compass.

[3] Watson D (2004). Match makers and deal breakers: Analyses of assortative mating in newlywed couples. J. Pers..

[4] Buss DM (1984). Marital assortment for personality dispositions: Assessment with three different data sources. Behav. Genet..

[5] Schwartz C, Graff N (2009). Assortative matching among same-sex and different-sex couples in the United States, 1990–2000. Demogr. Res..

[6] Robinson MR (2017). Genetic evidence of assortative mating in humans. Nat. Hum. Behav..

[7] Vandenberg SG (1972). Assortative mating, or who marries whom?. Behav. Genet..

[8] Epstein E, Guttman R (1984). Mate selection in man: Evidence, theory, and outcome. Biodemogr. Soc. Biol..

[9] Hitsch GJ, Hortaçsu A, Ariely D (2010). What makes you click?—Mate preferences in online dating. Quant. Mark. Econ..

[10] Watson D, Beer A, McDade-Montez E (2014). The role of active assortment in spousal similarity. J. Pers..

[11] Xie Y, Cheng S, Zhou X (2015). Assortative mating without assortative preference. Proc. Natl. Acad. Sci..

[12] Zajonc RB, Adelmann PK, Murphy ST, Niedenthal PM (1987). Convergence in the physical appearance of spouses. Motiv. Emot..

[13] Zajonc RB (1985). Emotion and facial efference: A theory reclaimed. Science.

[14] Schwartz CR (2010). Earnings inequality and the changing association between spouses’ earnings. Am. J. Sociol..

[15] Schwartz CR (2010). Pathways to educational homogamy in marital and cohabiting unions. Demography.

[16] Luo S (2009). Partner selection and relationship satisfaction in early dating couples: The role of couple similarity. Pers. Individ. Dif..

[17] Caspi A, Herbener ES (1993). Marital assortment and phenotypic convergence: Longitudinal evidence. Biodemogr. Soc. Biol..

[18] Feng D, Baker L (1994). Spouse similarity in attitudes, personality, and psychological well-being. Behav. Genet..

[19] Glicksohn J, Golan H (2001). Personality, cognitive style and assortative mating. Pers. Individ. Dif..

[20] Gonzaga GC, Carter S, Galen Buckwater J (2010). Assortative mating, convergence, and satisfaction in married couples. Pers. Relatsh..

[21] Caspi A, Herbener ES, Ozer DJ (1992). Shared experiences and the similarity of personalities: A longitudinal study of married couples. J. Pers. Soc. Psychol..

[22] Lemperle G, Holmes RE, Cohen SR, Lemperle SM (2001). A classification of facial wrinkles. Plast. Reconstr. Surg..

[23] Gillbert DT, Fiske ST, Lindzey G (1998). The Handbook of Social Psychology.

[24] Earley PC, Ang S (2003). Cultural Intelligence: Individual Interactions Across Cultures.

[25] Berger J (2016). Invisible Influence: The Hidden Forces that Shape Behavior.

[26] Niedenthal PM (2007). Embodying emotion. Science.

[27] Chartrand TL, Bargh JA (1999). The chameleon effect: The perception–behavior link and social interaction. J. Pers. Soc. Psychol..

[28] Hinsz VB (1989). Facial resemblance in engaged and married couples. J. Soc. Pers. Relat..

[29] Griffiths RW, Kunz PR (1973). Assortative mating: A study of physiognomic homogamy. Soc. Biol..

[30] Cao, Q., Shen, L., Xie, W., Parkhi, O. M. & Zisserman, A. VGGFace2: A dataset for recognising faces across pose and age. In *2018 13th IEEE International Conference on Automatic Face & Gesture Recognition (FG 2018)* 67–74 (IEEE, New York, 2018). 10.1109/FG.2018.00020.

[31] Parkhi, O. M., Vedaldi, A. & Zisserman, A. Deep face recognition. In *Procedings of the British Machine Vision Conference 2015* (eds. Xie, X., Jones, M. W. & Tam, G. K. L.) 41.1–41.12 (British Machine Vision Association, 2015). 10.5244/C.29.41.

[32] Kortylewski, A. *et al.* Empirically analyzing the effect of dataset biases on deep face recognition systems. In *2018 IEEE/CVF Conference on Computer Vision and Pattern Recognition Workshops (CVPRW)* 2174–217409 (IEEE, New York, 2018). 10.1109/CVPRW.2018.00283.

[33] Abel EL, Kruger ML (2011). Facial resemblances between heterosexual, gay, and lesbian couples. Psychol. Rep..

[34] Alvarez L, Jaffe K (2004). Narcissism guides mate selection: Humans mate assortatively, as revealed by facial resemblance, following an algorithm of “self seeking like”. Evol. Psychol..

[35] Wong YK, Wong WW, Lui KFH, Wong AC-N (2018). Revisiting facial resemblance in couples. PLoS ONE.

[36] Rhodes MG, Anastasi JS (2012). The own-age bias in face recognition: A meta-analytic and theoretical review. Psychol. Bull..

[37] Wiese H, Schweinberger SR (2018). Inequality between biases in face memory: Event-related potentials reveal dissociable neural correlates of own-race and own-gender biases. Cortex.

[38] Levay KE, Freese J, Druckman JN (2016). The demographic and political composition of mechanical turk samples. SAGE Open.

