# Supplementary material for: Spouses’ faces are similar but do not become more similar with time
Source: Sci Rep. 2020 Oct 12;10:17001. doi: 10.1038/s41598-020-73971-8 (PMC7550338; doi:10.1038/s41598-020-73971-8)
Supplement: Supplementary file 1 — Supplementary Information. [file 41598_2020_73971_MOESM1_ESM.doc]

# Supplementary Information for

Spouses’ faces are similar but do not converge with time.

Pin Pin Tea-makorn, Michal Kosinski

Correspondence to: [michalk@stanford.edu](mailto:michalk@stanford.edu)

Diversions from the Preregistered Research Protocol

The preregistered description of the *dependent variables* stated: “Facial similarity of spouses at marriage and at least 10 years later. Facial similarity of random pairs of men and women at marriage. A facial recognition system will be used to represent a face as a vector (which we will call face vector), and the facial similarity between a pair of faces will be evaluated from the Euclidean similarity of the face vectors.” While conducting the research, we introduced the following changes:

- As our sample contained fewer than 10 couples married for less than 20 years, we excluded those 10 couples.
- Euclidean similarity was replaced with cosine similarity, following the standard approach used in the field.^1^ The results are virtually identical regardless of whether Euclidean or cosine similarity is used.
- Following the feedback from the reviewers, we expanded the protocol by supplementing VGGFace2 with human judges (both produced similar results).
- Following the feedback from the reviewers, we have additionally collected human ratings of the couples’ likelihood of being married. No increase in the likelihood of being married was observed.

The preregistered description of the rules for excluding observations stated: “We will exclude photos [where] the facial recognition system cannot recognize the face. We will also exclude photos that have a resolution of the cropped face below 30 x 30 pixels.” While conducting the research, we introduced the following changes:

- We excluded faces smaller than 120 x 120 pixels and with an absolute value of yaw and pitch greater than 55° and 24°, respectively. After reviewing the first images collected by the research assistants, we realized that facial images smaller than 120 x 120 pixels and those in which the subjects face away from the camera are unlikely to contain enough information for judging facial similarity.

**References**

1. Cao, Q., Shen, L., Xie, W., Parkhi, O. M. & Zisserman, A. VGGFace2: A Dataset for Recognising Faces across Pose and Age. in *2018 13th IEEE International Conference on Automatic Face & Gesture Recognition (FG 2018)* 67–74 (IEEE, 2018). doi:10.1109/FG.2018.00020.

**Figure Legends**

**Fig. S1.** The distribution of spouses’ age (estimated using Face++) at the beginning of marriage and 20 to 69 years later.

**Supplementary Table S1. The Average Similarity Rank Between Spouses at Marriage and Later (the Lower the Rank, the More Similar the Faces)**

|  |  | Human judges | | | |  | VGGFace2 | | | |
| --- | --- | --- | --- | --- | --- | --- | --- | --- | --- | --- |
| Time span | N | At marriage | Later | t | p |  | At marriage | Later | t | p |
| All (20–69) | 517 | 2.75 [2.69, 2.81] | 2.89 [2.83, 2.96] | -3.70 | <.001s |  | 2.89 [2.76, 3.02] | 2.98 [2.86, 3.11] | -1.04 | .30 |
| 20–29 | 31 | 3.12 [2.86, 3.39] | 2.96 [2.67, 3.25] | .93 | .36 |  | 2.56 [2.01, 3.11] | 2.87 [2.33, 3.41] | -.98 | .33 |
| 30–39 | 21 | 2.89 [2.55, 3.22] | 2.80 [2.42, 3.18] | .38 | .71 |  | 2.88 [2.26, 3.50] | 3.33 [2.64, 4.02] | -.95 | .35 |
| 40–49 | 49 | 2.89 [2.72, 3.07] | 2.91 [2.70, 3.12] | -.16 | .87 |  | 3.15 [2.66, 3.64] | 3.06 [2.69, 3.44] | .37 | .71 |
| 50–59 | 306 | 2.67 [2.59, 2.75] | 2.92 [2.84, 3.00] | -4.93 | <.001 |  | 2.88 [2.71, 3.04] | 3.02 [2.85, 3.19] | -1.27 | .21 |
| 60–69 | 110 | 2.77 [2.64, 2.89] | 2.81 [2.67, 2.95] | -.51 | .61 |  | 2.91 [2.63, 3.19] | 2.80 [2.55, 3.06] | .58 | .57 |
| *Note.* Values in square brackets indicate 95% confidence intervals. Ranks’ test–retest reliability, defined as Spearman’s rank correlation between the ranks obtained for the stimulus sets featuring wives and husbands as targets, equaled ρ=0.33 for the human judges and ρ=0.54 for VGGFace2. The correlation between the ranks produced by human judges and VGGFace2 equaled ρ=0.26. Human judges’ inter-rater reliability, , defined as the average Kendall rank correlation between two randomly selected rankings for each stimulus set, equaled τ = 0.16. | | | | | | | | | | |

**Supplementary Table S2.** The Average Marriage Likelihood Rank Between Spouses at Marriage and Later (the Lower the Rank, the More Likely the Pair Are Married)

|  |  | Human judges | | | |
| --- | --- | --- | --- | --- | --- |
| Time span | N | At marriage | Later | t | p |
| All (20–69) | 517 | 3.24 [3.20, 3.28] | 3.28 [3.24, 3.32] | -1.51 | .13 |
| 20–29 | 31 | 3.21 [3.02, 3.40] | 3.16 [3.00, 3.32] | .49 | .63 |
| 30–39 | 21 | 3.42 [3.20, 3.66] | 3.10 [2.87, 3.32] | 2.11 | .047 |
| 40–49 | 49 | 3.36 [3.19, 3.53] | 3.23 [3.11, 3.35] | 1.49 | .14 |
| 50–59 | 306 | 3.23 [3.18, 3.28] | 3.30 [3.25, 3.35] | -2.12 | .035 |
| 60–69 | 110 | 3.20 [3.12, 3.27] | 3.31 [3.26, 3.55] | -2.02 | .045 |
| *Note.* Values in square brackets indicate 95% confidence intervals. Ranks’ test–retest reliability, defined as Spearman’s rank correlation between the ranks obtained for the stimulus sets featuring wives and husbands as targets, equaled ρ=0.11. Human judges’ inter-rater reliability, defined as the average Kendall rank correlation between two randomly selected rankings for each stimulus set, equaled τ=0.025. | | | | | |
