# Supplementary figures and images for: Spouses’ faces are similar but do not become more similar with time
Source: Sci Rep. 2020 Oct 12;10:17001. doi: 10.1038/s41598-020-73971-8 (PMC7550338; doi:10.1038/s41598-020-73971-8)

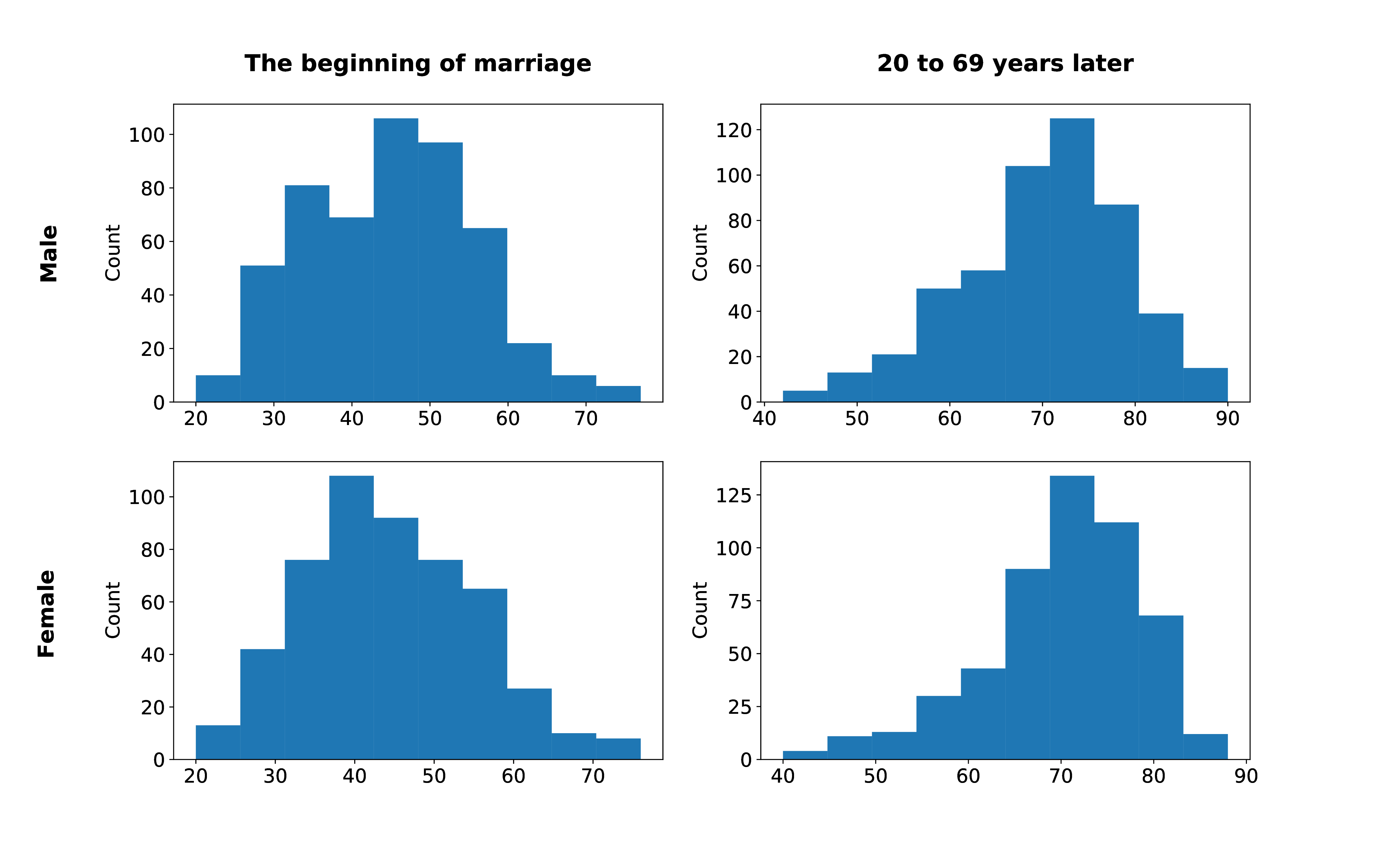

Supplement: Supplementary file 2 — Supplementary Figure S1. [file 41598_2020_73971_MOESM2_ESM.tiff]
